# Introduction and overview on Autogenous Platelet Concentrates

**DOI:** 10.1111/prd.12607

**Published:** 2024-09-11

**Authors:** Marc Quirynen, Anton Sculean, Juan Blanco, Hom‐Lay Wang, Nikos Donos

**Affiliations:** ^1^ Department of Oral Health Sciences, KU Leuven & Dentistry (Periodontology) University Hospitals Leuven Leuven Belgium; ^2^ Department of Periodontology University of Bern Bern Switzerland; ^3^ Department of Surgery (Stomatology, Unit of Periodontology) Universidade de Santiago de Compostela Santiago de Compostela Spain; ^4^ Department of Periodontics and Oral Medicine The University of Michigan, School of Dentistry Ann Arbor Michigan USA; ^5^ Institute of Dentistry, Centre for Oral Clinical Research, Barts and The London School of Medicine and Dentistry Queen Mary University of London (QMUL) London UK

**Keywords:** alveolar ridge preservation, ARP, autogenous platelet concentrates, APC, GBR, GTR, guided bone regeneration, guided tissue regeneration, intra‐bony defects, medication‐related osteonecrosis of the jaw bones, MRONJ, plastic periodontal surgery, sinus floor elevation (lateral window technique, transcrestal technique)

## Abstract

This special issue on autologous platelet concentrates (APCs) provides clinicians with an overview on the current understanding of the use of these biomaterials for soft and hard tissue regeneration. The included papers summarize scientific evidence and the clinical findings, presented in simple tables that outline potential benefits including Patient Reported Outcome Measures (PROMs). This approach enables clinicians to assess clinical relevance and researchers to identify significant gaps in the literature. The first part provides a comprehensive summary of the basic science surrounding APC, with particular focus on their preparation methods. Clear recommendations are outlined, which are crucial for obtaining high‐quality APCs, alongside an exploration of how APCs may influence both soft and hard tissue healing processes. Part 2 delves into the clinical evidence for the potential benefits of APCs across a range of applications: alveolar ridge preservation, sinus floor elevation, periodontal plastic surgery, guided tissue regeneration, guided bone regeneration, the healing of Medication‐Related Osteonecrosis of the Jaw (MRONJ), and endodontic surgery. In the part 3, the discussion turns to the effects of APCs on the healing of extra‐oral wounds, including diabetic foot ulcers, venous leg ulcers, pressure injuries, burns, and more. The fourth section offers a detailed, step‐by‐step flowchart for each treatment modality, providing a clear guide for clinical application.

## 1 GENERAL INTRODUCTION

Autologous Platelet Concentrates (APCs) represent a category of bioactive additives derived from patient's own blood through a chair‐side centrifugation process. They aim to accelerate and promote natural healing and regeneration of both soft and hard tissue by concentrating platelets, leucocytes, and other therapeutic constituents of blood such as fibrinogen/fibrin, growth factors, cytokines, and circulating cells, *at the site of surgery*.

APCs are a subset of autologous blood‐derived products (ABP), which also include other biologic agents like enamel matrix derivative, recombinant human platelet‐derived growth factor BB, and recombinant human bone morphogenetic protein 2.^1^

Over the past three decades, four main types of APCs have been introduced, which can be categorized into two broad classifications based on their physical characteristics and content: Platelet‐Rich Plasma types (PRP) representing liquid platelet suspensions that can be transformed into a fibrin gel after activation, and Platelet‐Rich Fibrin types (PRF) which form solid fibrin clots because of a highly polymerized three‐dimensional (3‐D) fibrin network.

These APCs are further divided into the following four families based on their leukocyte content and fibrin structure, as detailed in the literature:

- Pure Platelet‐Rich Plasma (P‐PRP) without leukocytes and with a low‐density fibrin network (e.g. plasma rich in growth factors (PRGF)),
- Leukocyte‐ and Platelet‐Rich Plasma (L‐PRP), also containing leukocytes (the amount depends on the protocol) but with a low‐density fibrin network (most other PRPs),
- Pure Platelet‐Rich Fibrin (P‐PRF) without leukocytes and with a high‐density fibrin network (infrequently used),
- Leukocyte‐ and Platelet‐Rich Fibrin (L‐PRF) with leukocytes (the amount depending on the protocol) and a high‐density fibrin network, or an injectable, flowable subcategory.^2^, ^3^, ^4^, ^5^, ^6^

**FIGURE 1:**
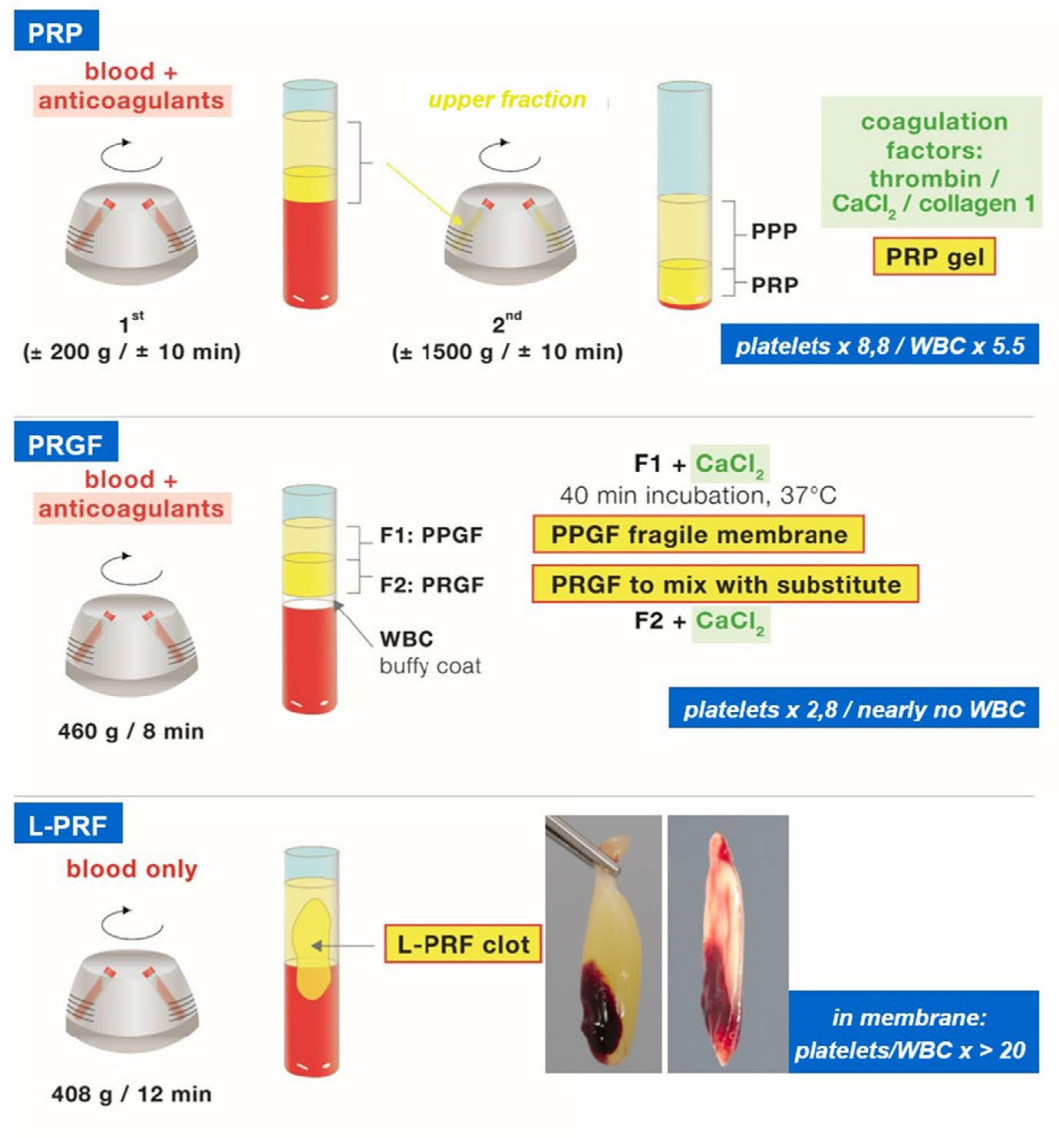
Differences in preparation between first (PRP, PRGF) and second (L‐PRF) generation APCs, with their increased concentration in platelets and leucocytes when compared to whole blood. L‐PRF, leukocyte‐ and platelet‐rich fibrin family; PPGF, plasma poor in growth factors; PPP, platelet‐poor plasma; PRGF, plasma rich in growth factors; PRP, platelet‐rich plasma; WBC, (white blood cells). (updated from Quirynen et al.^12^)

The first generation of APCs, PRP, and PRGF emerged in the late 1990s.^7^, ^8^, ^9^ Their preparation is relatively complex, often including the use of anticoagulants and coagulation factors (Figure 1). In contrast, the second‐generation APCs, L‐PRF, introduced by Choukroun et al in 2001, is easier to prepare and does not require the use of neither anticoagulants nor coagulation factors, ensuring the final product is 100% autogenous (for more details see Quirynen et al. 2024).^10^, ^11^ More recently several light modifications in the preparation of L‐PRF have been introduced including: concentrated growth factors (CGF), advanced PRF (A‐PRF), advanced plus PRF (A‐PRF+), titanium‐prepared platelet rich‐fibrin (T‐PRF), platelet‐rich fibrin prepared with a horizontal centrifuge (H‐PRF).

This special issue aimed to delve into the basic science to better understand the mechanisms via which APCs can affect wound healing and repair. It is also intended to offer an up‐to‐date, evidence‐based perspective on the therapeutic use of APCs without commercial interference.

## 2 BASIC SCIENCE ON APCs

### 2.1 APCs and wound healing

Wound healing is a complex and interactive process that encompasses a range of cellular and biologic processes. These processes are intricately regulated to allow optimal repair and regeneration of damaged tissues. Bartold and Ivanovski (2024) have summarized the current understanding of the factors and processes involved in both soft and hard tissue healing, including the cellular and biological mediators, and made specific extrapolations to periodontal tissues regeneration.^13^ Furthermore, they shed light on the potential pathways through which APCs could play a role in tissue healing.

### 2.2 Differences between first and second‐generation APCs

The first‐generation APCs are significantly different from the second‐generation APCs. Besides their large difference in the concentration of platelets and leukocytes (Figure 1), they also differ in the rate of growth factor release, antibacterial capacity, physical characteristics of their end product (gel for PRP, weak membrane for PRGF, strong 3D fibrin network for L‐PRF membrane) which explains partially the variability in their clinical outcome. The fibrin matrix of L‐PRF traps platelets, leukocytes, cytokines, and circulating stem cells leading to a long‐term release of growth factors including platelet derived growth factor (PDGF‐ββ), transforming growth factor (TGF‐β1), vascular endothelial growth factor (VEGF), insulin‐like growth factor, cytokines (interleukin 1β, IL‐6, IL‐4), and tumor necrosis factor. These aspects are discussed in detail by Calciolari and co‐workers (2024).^6^

### 2.3 Potential explanations for the beneficial effects of APCs (PRP, PRGF as well as L‐PRF)

In the paper of Gruber (2024), the protocols and tools used to prepare PRP are analyzed.^14^ This paper examined the cellular and molecular composition of PRP, with particular emphasis on platelets, leucocytes, and the fibrin‐rich extracellular matrix found in coagulated plasma. He also provided an overview of the potential beneficial effects of PRP on a cellular and molecular level and summarizes its current use in dentistry and other medical fields.

Blanco et al. (2024), also featured in Perio 2000, delve into the mechanisms behind Leukocyte‐ and Platelet‐Rich Fibrin (L‐PRF).^15^ Their work provides a comprehensive description of the molecular components within L‐PRF. Additionally, they review the supporting evidence for its effects on anti‐inflammatory response, pain relief, hemostasis, and antimicrobial action. The article elucidates the biological mechanisms through which L‐PRF can influence bone/soft tissue regeneration.

### 2.4 Critical aspects for the preparation of APCs

To obtain high‐quality APC, the centrifugation protocol is essential, including factors such as: (centrifuge radius, centrifugation time and speed, inner characteristics of blood tubes, timing during the preparation, etc).^11^ Quirynen and co‐workers (2024) prepared recommendations for an optimal centrifugation. More recently, several light modifications in the way L‐PRF has to be prepared have been introduced including: (CGF = concentrated growth factors, A‐PRF = advanced PRF, A‐PRF+ = advanced plus PRF, T‐PRF = titanium‐prepared platelet‐rich‐fibrin, H‐PRF = platelet‐rich fibrin prepared with a horizontal centrifuge).^11^ The differences between these modifications are also outlined in this paper.

Despite the modifications, all these L‐PRF forms share common features: high concentrations of leukocytes and platelets, enmeshed within a dense fibrin network, following a similar preparation protocol with variations in centrifugal force, time, and the type of blood collection tube, whether using a fixed‐angle or horizontal centrifuge. Given that the current data suggest only minor differences in clinical outcomes among these variants, this special issue refers to them collectively as the L‐PRF family, except in the detailed tables of individual papers that provide a summary of the clinical studies.

## 3 INTRA‐ORAL APPLICATIONS OF APCs

Ten systematic reviews, some of which incorporate meta‐analyses, have rigorously examined the scientific evidence supporting the use of APCs during periodontal surgery. Each review focused on answering the question “*Can APCs improve the outcome of a specific intervention?*”. Furthermore, they sought to identify the most optimal condition/treatment option, taking into account both patient reported outcome measures (PROMs) and clinical outcome.

### 3.1 Can APCs reduce bone resorption and/or improve PROM after tooth extraction?

**FIGURE 2:**
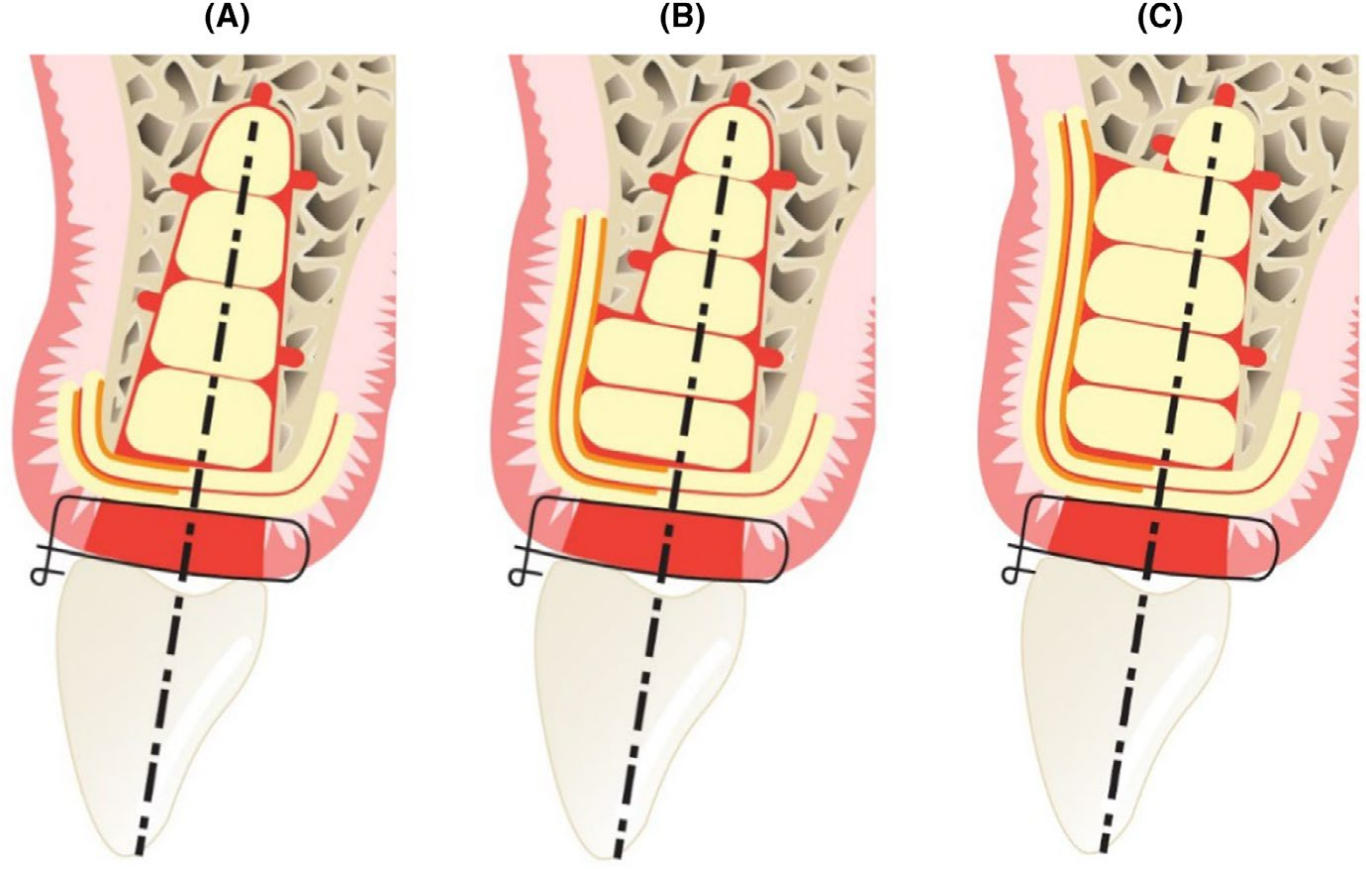
Treatment strategies for the application of a second‐generation platelet concentrate during alveolar ridge preservation (ARP), dependent on presence/degree of a buccal bone dehiscence. In fact for intact sockets (A), sockets with a small buccal bone defect (B), or for those with a large defect (C), a similar approach has been used in most papers. The socket has to be filled with L‐PRF plugs, membranes, well condensed. A healing by secondary intention is preferred, in order not to influence the vestibulum depth, applying an L‐PRF membrane. This will simplify the intervention. In case of a large bony defect (C), one can choose to wait for a soft‐tissue healing and afterwards perform a GBR. (Structure in yellow with orange line represents an L‐PRF membrane (in orange their face part), the more oval structures are L‐PRF plugs).

The adjunctive benefit when adding APCs to extraction sockets was examined in a systematic review by Siawasch and co‐workers (2024).^16^ Typically, APCs were mostly applied immediately after tooth extraction, with healing predominantly occurring by secondary intention (Figure 2). In some instances, the socket was covered with an autologous fibrin membrane to aid closure. In case of a larger bony dehiscence, one can of course opt for an APC application after soft tissue healing.^17^

Short‐term studies have investigated soft tissue healing and patient‐reported outcome measures (PROMs), including pain, swelling, incidence of post‐extraction bleeding, and occurrence of alveolitis. Conversely, long‐term research has concentrated on the evaluation of alveolar bone resorption in both horizontal and vertical dimensions, as well as the extent of socket fill, and the quality and density of the new bone. These studies have compared outcomes following the use of autologous platelet concentrates (APCs) to those of natural, unaided healing, particularly in class 1 and class 2 bony defects. A few randomized controlled trials (RCTs) have assessed the effectiveness of an APC in comparison with other biomaterials such as hydroxyapatite, bioactive glass, bovine bone mineral, and freeze‐dried bone allograft.

Additionally, Siawasch and colleagues (2024) directed their research efforts, as presented in Perio 2000, toward the impact of APCs on the healing process after the extraction of impacted mandibular third molars. This systematic review primarily focused on PROMs and the subsequent quality of the regenerated bone.^18^

### 3.2 Can APCs facilitate bone regeneration after sinus floor elevation?

**FIGURE 3:**
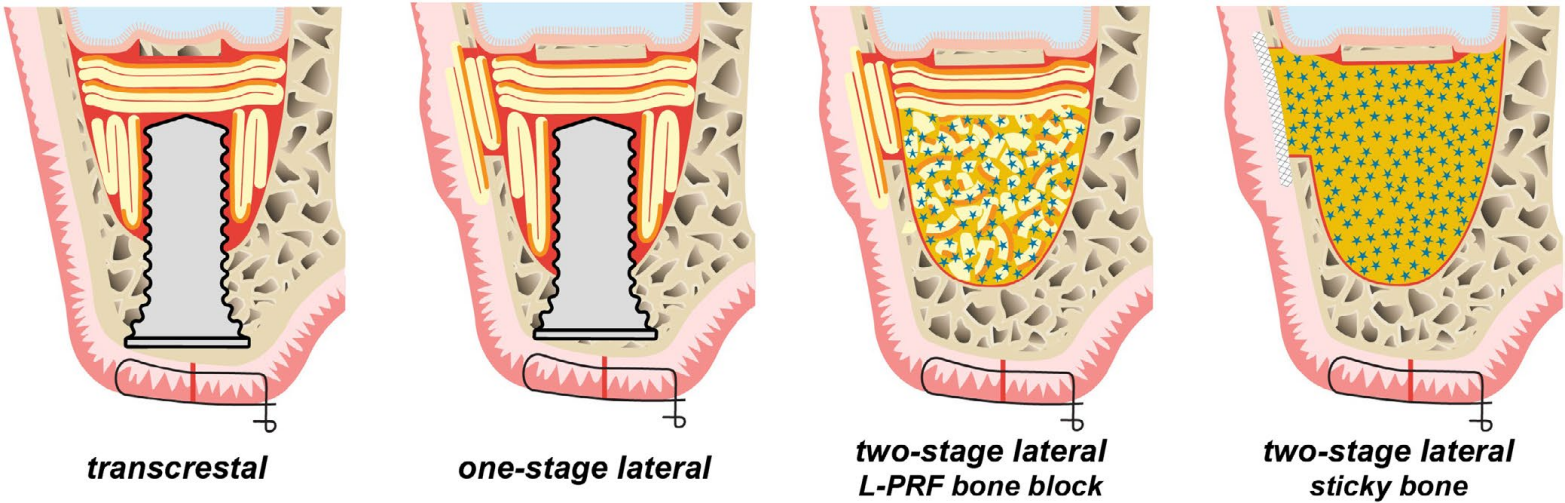
Treatment strategies for the application of an APC after sinus floor elevation: Transcrestal approach using a PRGF fibrin clot or L‐PRF membranes, one‐stage lateral window applying L‐PRF membranes in the augmented area, eventually also in and over the bony window, two‐stage lateral window using either an L‐PRF bone‐block or sticky bone (for the latter all APCs are useful). (Structure in yellow with orange line represents an L‐PRF membrane (in orange their face part), blue stars are particles of bone substitute (embedded in a flowable APC in light orange), eventually including chopped pieces of L‐PRF membrane; the white structure with cross hatch in two directions) represents a barrier membrane).

This question is answered by Valentini and co‐workers (2024).^19^ The application of an APC depends on the treatment strategy (Figure 3). For a transcrestal approach, the authors identified two papers where PRGF plugs were used, and 11 papers where L‐PRF membranes were applied as the sole grafting material. For a one‐stage lateral window approach that employs APCs solely as the grafting material, only papers with the use of L‐PRF membranes could be retrieved. In such scenarios, it is a *conditio sine qua non*, that implant(s) are present to act as “tent poles” supporting the elevated Schneiderian membrane, and that the fibrin membranes are strong. For a two‐stage lateral window, APC matrices are not recommended due to their rapid resorption. Instead, APCs may be utilized in conjunction with a bone substitute to enhance and expedite bone regeneration. For this purpose, all three types of APCs have been tested. The authors also evaluated whether PRGF or L‐PRF membranes could be used to seal membrane tears and/or to close the lateral window.

### 3.3 Can APCs facilitate alveolar bone regeneration (horizontal: simultaneous/staged & vertical)?

**FIGURE 4:**
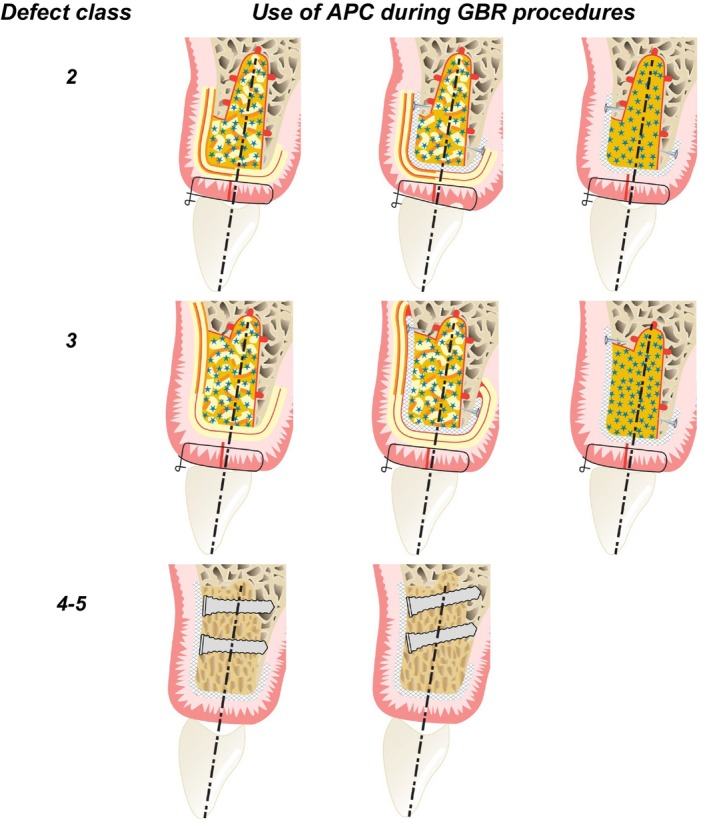
Options for the use of an APC during alveolar bone regeneration, depending on the defect classification of Benic and Hämmerle.^20^ Class 2: In case of a well‐contained defect, most clinicians do not use a barrier membrane, only an autologous fibrin membrane (made with PRGF or L‐PRF); for non‐contained defects, a barrier membrane is often added. An APC (flowable form can be used to prepare sticky bone), now covered with barrier membrane. For class 3 defects, a barrier membrane reduces the risk for a graft collapse. For class 4 and 5 defects (with the need of a vertical augmentation), often a block graft covered with a barrier membrane is applied. For these bone defects, APCs can be used to soak the graft (faster bone formation), or to cover the barrier membrane (supporting the soft‐tissue healing in case of a wound dehiscence). (Structure in yellow with orange line represents an L‐PRF membrane (in orange their face part), blue stars are particles of bone substitute (embedded in a flowable APC in light orange), eventually including chopped pieces of L‐PRF membrane; the white structure with cross hatch in two directions) represents a barrier membrane).

Numerous techniques have been described to reconstruct deficient alveolar ridges. It is important to differentiate among various procedures: firstly, between a one‐stage procedure, where augmentation and implant placement occur simultaneously, and a two‐stage approach, where augmentation precedes implant placement at a later time. Additionally, a distinction should be made between horizontal and vertical augmentations, with the latter being notably more challenging, as well as between the dimensions of the bone defect (for example, as classified by Benic and Hammerle 2014),^20^ and between contained and non‐contained defects. Blanco and co‐workers (2024) reviewed the benefits of using APCs during these interventions, taking into consideration all the above‐mentioned conditions, and identified a large variety in treatment options (Figure 4).^17^ The hypothesis is that APCs, with their release of growth factors and fibrin network, could facilitate/speed up the healing process. Furthermore, the review also considered the potential benefits of using APCs in combination with other biomaterials.

### 3.4 What are the benefits of APCs in the treatment infra‐bony defects?

**FIGURE 5:**
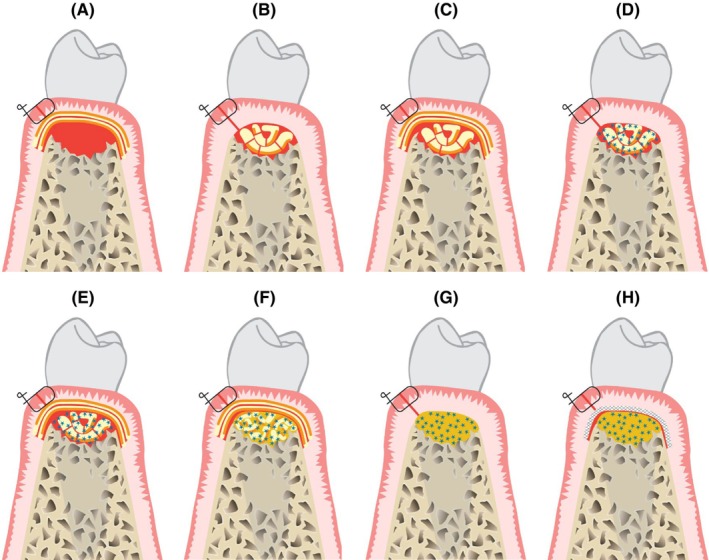
A large variety in APC applications have been explored, ranging from, of course after optimal debridement and surface conditioning: (A) using an autologous fibrin membrane only to cover the bony defect, filled with a blood clot, (B) filling of the bony defect with an APC, (C) filling of the defect combined with its' coverage using an autologous fibrin membrane, (D) a combination of an APC with a bone substitute to fill the defect, (E) the same combination, but now also sealed with an autologous fibrin membrane, (F) the same approach but applying an L‐PRF bone block, (G) the use of sticky bone to fill the defect, (H) the previous approach, now covered with a barrier membrane. (Structure in yellow with orange line represents an L‐PRF membrane (in orange their face part), blue stars are particles of bone substitute (sometimes embedded in a flowable APC in light orange), eventually including chopped pieces of L‐PRF membrane; the white structure with cross hatch in 2 directions represents a barrier membrane).

A substantial body of research has investigated the adjunctive benefits of applying APCs to intra‐bony defects during an open flap debridement. The literature review elucidated various strategies for employing APC: covering the defect, filling it, or combining both techniques. These can be applied either as the sole biomaterial or in combination with a bone substitute, as illustrated in Figure 5. Miron and co‐authors (2024 a,b) in their contributions to Perio 2000, conducted two separate meta‐analyses in a pair of papers.^21^, ^22^ Their reviews assessed the clinical efficacy of these strategies on parameters such as PROM, fill of bony defects, probing pocket depth reduction, and improvements in clinical attachment level. One paper focused on defects surrounding teeth, while the other was dedicated to furcation defects.

### 3.5 What are the benefits of APCs in plastic periodontal surgery?

APCs are known to support the soft tissue healing as demonstrated by their impact on the healing of extra‐oral wounds.^23^, ^24^ Barootchi and co‐workers (2024) reviewed the adjunctive benefit of adding APCs during a coronally advanced flap (CAF) procedure.^25^ A significant number of RCTs explored the insertion of L‐PRF membranes or PRGF gels, between the CAF and the root surface, in comparison to CAF alone. In other studies, a liquid PRP was used to condition the root surface or to soak a collagen sponge. Another series of studies compared the use of L‐PRF membranes or PRGF gels toward a connective tissue graft (CTG). The authors considered besides clinical parameters such as root coverage, probing depth, attachment gain, and also PROMs, particularly relevant when a CTG is.

### 3.6 What are the benefits of APCs in the osseointegration process (biomimetics)?

The establishment of a stable fibrin clot immediately following implant placement is essential in the early phase of osseointegration, providing a provisional scaffold for the migration of differentiating osteogenic cells toward the implant surface. To enhance or facilitate this process, a variety of substrates including extracellular matrix components, custom‐designed peptides, and growth factors have been proposed for use as a potential biological adjunct on oral implants. Although biomimetic strategies (in which the designs, systems, and elements of nature are emulated to address complex human challenges) for the functionalization of implant surfaces, such as coating with specific peptides during manufacture, are viewed as promising, they are not yet available for clinical application. Ivanovski and co‐workers (2024) reviewed whether APCs have the potential to enhance this process by modifying the interface between the host and the surface of the titanium implant.^26^ Different approaches have been tested: (a) dipping the implant in a flowable APC, (b) wrapping the implant immediately before insertion in an APC membrane/gel, (c) applying an APC into the osteotomy before implant insertion, (d) covering the implant and surrounding bone with an APC membrane/gel, or a combination of the previous techniques. To answer the question whether APC could play a beneficial role in the osteointegration process the authors considered laboratory studies, animal trials, as well as clinical studies.

### 3.7 What are the benefits of APCs in the treatment of MRONJ?

Antiresorptive drugs or antiangiogenic agents are usually administered at a high dose in the prevention and treatment of bone metastasis and other malignant conditions, while lower doses are used in the management of osteoporosis, Paget disease, and symptomatic fibrous dysplasia. Patients treated with these medications may experience a medication‐related osteonecrosis of the jaw (MRONJ). Bennardo and co‐workers (2024) reviewed the literature to explore the potential benefits of adjunctive APCs during standard treatment protocols for MRONJ.^27^ The addition of APCs might be anticipated to enhance healing due to several factors: the elevated concentration of platelets (and leucocytes), the release of important growth factors (which are involved in collagen production, anti‐inflammatory action, initiate vascular growth, induce cell differentiation, control local inflammatory responses, and thereby facilitate tissue healing/regeneration), and their antibacterial capacity. This paper thoroughly examined the clinical evidence supporting the use of APCs and included recommendations for how to apply APCs in the treatment of MRONJ.

### 3.8 What are the benefits of APCs in endodontic surgery?

Sabeti and co‐workers (2024) reviewed the literature on the beneficial effects of adding APC during endodontic surgery.^28^ They made a distinction between pure apical lesions, and endo‐perio, perio‐endo, or true combined defects. By reviewing the literature, they observed varying degrees of benefits when using APCs for the above‐mentioned pathologies.

**Table:** 

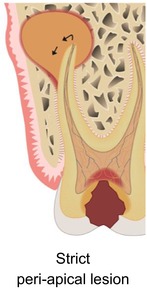	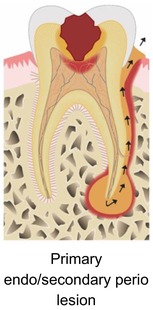	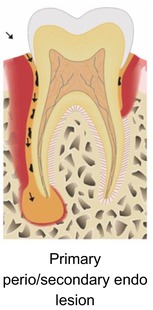	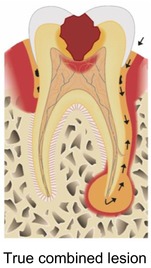
Strict peri‐apical lesion	Primary endo/secondary perio lesion	Primary perio/secondary endo lesion	True combined lesion

## 4 EXTRA‐ORAL APPLICATIONS OF APCs

### 4.1 What are the benefits of APCs in the healing of extra‐oral chronic/demanding wounds

Chronic wounds fail to progress within a specific timeframe despite appropriate care due to an inability to proceed through the normal stages of healing. The normal physiology is transformed into the pathophysiology of a chronic cycle, without a distinct wound closure endpoint. The hypothesis is that APCs could improve the healing of chronic wounds because of their increased concentration of platelets and leucocytes, the release in growth factors and cytokines crucial for wound healing/repair, the antibacterial capacity, etc.^6^, ^13^, ^14^, ^15^

Perussolo and co‐workers (2024) reviewed the benefits of applying PRP and PRGF into chronic wounds, and Pinto and co‐workers (2024) reported the benefits when using matrices from the L‐PRF family.^23^, ^24^ Both reviews explored their use either as alternative for or as adjunctive to conventional/standard treatment for various extra‐oral wounds and ulcers (e.g., diabetic foot ulcers, venous leg ulcers, pressure ulcers, Leprosy ulcers, as well as other very demanding acute wounds and burns). They also included a series of clinical cases illustrating their impact. In addition to aspects such as wound closure, both papers concentrate on PROMs, encompassing patient quality of life, healing time, recurrence of the wound, pain management, treatment costs, analgesics requirements, and potential complications. To help clinicians, Pinto and co‐workers (2024) also included recommendations for the use of L‐PRF in the treatment of extra‐oral wounds.^24^

### 4.2 What are the benefits of APCs in facial aesthetics

APCs are gaining traction within the realm of facial aesthetic treatments. Miron and co‐workers (2024) elucidate the multifaceted applications of APCs in aesthetic therapy which include dermal procedures such as microneedling, intradermal injection, skin regeneration around the eye (peri‐orbital), and mouth (peri‐oral) as well as their use as volumizing substance.^29^ APCs can be administered either alone or synergistically with other materials such as hyaluronic acid or nano fat. Their comprehensive review delves into the evidence‐based benefits of these applications. It also provides insight into the longevity of the treatment's effects in contrast to alternative substances, alongside discussing PROMs. Further enriching the review, a collection of illustrative videos demonstrates the various treatment techniques in practice, offering a visual aid to the described procedures.

## 5 INSTRUCTIONS FOR THE OPTIMAL USE OF THE SECOND‐GENERATION PLATELET CONCENTRATES

Owing to the fact that the majority of clinical trials focused on second‐generation APCs, and given that these second‐generation APCs offer added benefits when compared to the first‐generation APCs the (co)‐editors of this special issue have opted to feature these advanced APCs for the formulation of their instructions.^30^

Considering an exhaustive review of the available clinical data and the extensive clinical expertise of the contributing authors, comprehensive recommendations have been crafted. These elaborate not only on the methods for preparing leukocyte‐ and platelet‐rich fibrin (L‐PRF)—both in its solid form and its liquid variants—but also on their application across diverse procedures. These include alveolar ridge preservation, management of intra‐bony defects, recession coverage, sinus floor elevation (encompassing both the lateral window technique and the transcrestal approach), and lateral bone augmentation.

Supplementary to these recommendations, a series of instructional videos are available, providing a dynamic means to better comprehend the procedural flowcharts.

**Table:** 

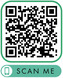	*For more information on the use of L‐PRF matrices in the above mentioned clinical indications you may wish to visit a webpage from Marc Quirynen with short videos summarizing the different procedures. Just scan this QR code. Following videos are available: alveolar ridge preservation, sinus floor elevation, lateral bone augmentation (GBR), open flap debridement combined with infra‐bony defect fill*. *You can also use this URL*: https://www.l‐prf4all.com/lprfperio2000additionalvideos

## CONFLICT OF INTEREST STATEMENT

All (co)‐authors declare that they have no conflict of interest in relation to this chapter, even though they might have received research support from different companies including: BioHorizon Inc., Bti, Camlog, Dentsply Sirona, Geistlich, Hu‐Frieddy, Henry Schein, Straumann, TiCare, DENTAID, Straumann, Geistlich, Regedent, Botiss, Stoma.

## Data Availability

Data sharing is not applicable to this article as no new data were created or analyzed in this study.
